# Pain, Clinical Tests, and Disability in Individuals With Radicular Low Back Pain: A Responsiveness and Correlation Study

**DOI:** 10.1155/prm/9995879

**Published:** 2026-05-22

**Authors:** Halidadjiyah, Prasert Sakulsriprasert, Wunpen Chansirinukor

**Affiliations:** ^1^ Faculty of Physical Therapy, Mahidol University, Nakhon Pathom, Thailand, mahidol.ac.th; ^2^ Institute of Health and Science Technology Wiyata Husada Samarinda, Kota Samarinda, Kalimantan Timur, Indonesia

**Keywords:** function, gait, radiculopathy, referred pain, sensitivity

## Abstract

**Background:**

This study aimed to investigate the responsiveness of pain, clinical tests, and disability in individuals with RLBP.

**Methods:**

Thirty‐two patients with RLBP were assessed for pain intensity (at rest and at worst), clinical tests comprising straight leg raising (SLR) and five‐meter walk test (5MWT), and disability was assessed by the Modified Oswestry Disability Index (MODI) at baseline, immediately after, 1 week, and 2 weeks after first‐session interventions. Change scores and effect size (ES) were calculated.

**Results:**

Pain at rest and pain at worst were most responsive with large ES, especially at 2 weeks (ES = 3.6 and 3.6, respectively). SLR and 5MWT were responsive with large ES at 2 weeks (ES = 0.9 and 0.9, respectively). Also, MODI was responsive at 2 weeks (ES = 1.0).

**Conclusions:**

This study indicated that the responsiveness of pain intensity (at rest and at worst), SLR, 5MWT, and MODI in patients with RLBP at 2 weeks after therapeutic interventions was evident.

## 1. Introduction

According to the diagnostic triage for low back pain (LBP), radicular LBP (RLBP) is one of three categories and accounts for approximately 5%–10% of all LBP patients [1, 2]. RLBP is a musculoskeletal condition that affects both men and women and is a frequent reason for seeking therapeutic intervention. The cardinal symptoms typically include LBP accompanied by radiating pain along the lower extremity, a condition referred to as radiculopathy. Patients with RLBP often describe these radiating sensations as electric shocks, burning, or sharp pain, which are attributable to irritation or compression of specific spinal nerves [3–5]. In addition, patients with RLBP also experience the alteration of motor function depending on the severity of nerve compression [6], increased work‐related disability, and a poorer prognosis compared to those with non‐RLBP [7].

Beyond spinal structural displacement and nerve root compression, disc herniation is a primary etiology of RLBP, typically necessitating confirmation via magnetic resonance imaging (MRI) [8]. Essential physical examination components include a comprehensive pain assessment to quantify symptom intensity, the straight leg raising (SLR) test to evaluate nerve root irritation, and the five‐meter walk test (5MWT) as an objective measure of functional mobility. Together with disability assessments, these tools provide a diagnostic and performance‐based baseline for monitoring daily physical capacity [6].

Management of RLBP includes both conservative and surgical approaches, with guidelines prioritizing an initial trial of nonoperative care, specifically, patient education, maintaining activity, and manual therapy [9–11]. These interventions aim to reduce pain, centralize symptoms, and restore functional capacity [12, 13]. Most patients respond well to conservative management, with significant recovery in pain, clinical markers, and disability often observed within 2 weeks for acute or subacute cases [8, 14]. Multimodal conservative care led to substantial improvements in pain, function, and mental health at 6‐month follow‐up. Epidural injections and physical therapy proved to be safe and effective, with a low 8% requirement for further intervention [15]. Neural mobilization is a well‐supported conservative technique that can meaningfully reduce radicular pain and disability, especially when combined with standard physiotherapy and exercise [16].

Responsiveness is a critical psychometric attribute that denotes an outcome measure’s ability to detect meaningful shifts in a patient’s health status [17]. While pain, disability (Modified Oswestry Disability Index [MODI]), and functional tests have demonstrated responsiveness within 2 weeks in nonspecific LBP (NSLBP) [18, 19], the distinct clinical characteristics of RLBP may lead to different outcomes. Currently, evidence regarding the responsiveness of pain, SLR, and 5MWT in RLBP populations is lacking. Therefore, this study aims to investigate the responsiveness of these clinical and functional measures over a 2‐week interval.

## 2. Methods

### 2.1. Study Design and Subjects

This was a cross‐sectional study based on data collected from our main study, which has already been published [14]. Data from 32 out of 58 patients were eligible for inclusion in the study. These patients were diagnosed with RLBP or lumbar radiculopathy by physicians at PKU Muhammadiyah Hospital, Yogyakarta, Indonesia, between December 2020 and January 2021. The inclusion criteria are as follows: age between 25 and 55 years, having LBP with unilateral radiating leg pain, intensity of pain at the low back area at least 3/10 on visual analog scale (VAS), having lumbar disc herniation (LDH) Grade 1 or Grade 2 confirmed by MRI, and characterized in 1–5 derangement classification [20], The exclusion criteria were as follows: obesity (body mass index at least 30 kg/m^2^), having specific spine disorders or red flags, pregnancy, spinal or leg surgery within 6 months, currently taking pain medication, and radicular symptom duration of more than 3 months and neurological signs (motor weakness, diminished deep tendon reflexes, or sensory loss), which are significant confounders influencing the recovery [21]. Written informed consent was obtained from all patients before participation. This study has been approved ethically in accordance with the Declaration of Helsinki by the Mahidol University–Central Institutional Review Board (MU‐CIRB), numbered MU‐CIRB 2020/170.2010.

### 2.2. Outcome Measures

Pain intensity was the primary outcome, which was measured with VAS for pain at rest and pain at worst, which is a 10‐cm horizontal line, the left end labeled with 0 “no pain” and the right end labeled with 10 “the most severe pain.” VAS is valid and reliable for LBP assessment [22]. The minimal clinically important difference (MCID) for LBP is 2.25 cm [23].

Clinical tests: there were 2 tests: SLR and 5MWT. The SLR was used to assess the neurodynamics of the leg. In this study, the SLR was assessed with the iHandy Level smartphone application in supine lying. The assessor raised the tested leg by supporting the ankle with the knee fully straight until the participant felt any symptoms present from the back that radiated to the leg. After calibration, the iHandy Level smartphone application was placed on the anterior aspect of the mid‐tibia. The minimal detectable change (MDC) for SLR is 5.7° [24].

5MWT: the participants were asked to walk between the two 5‐meter points at their preferred walking speed. If the participants could not continue or they felt any symptoms or discomfort, they could stop. A familiarization trial was allowed before the assessment. The MCID for gait speed is 0.1 m/s [25]. The gait speed is convertible to the 5MWT. Therefore, the MCID for the 5MWT is 0.5 s.

Disability: the MODI, the Indonesian version [26], was used to assess the disability level at baseline and at the end of the program. The MODI is reliable and valid for LBP assessment [27–29], with the MCID of 9 points [23].

### 2.3. Study Procedure

The participants were assessed at four time points: baseline, immediately after the initial intervention (postimmediate), 1 week after the initial intervention (post‐1‐week), and 2 weeks after the initial intervention (post‐2‐week). The outcome measures included pain intensity (at rest and at worst), clinical tests including the SLR test and the 5MWT, and disability (MODI). All outcome measures, except disability, were evaluated at each of the four assessment points. The level of disability, as measured by the MODI, was assessed only at baseline and at the post‐2‐week time point. Regarding interventions, participants received one of two physical therapy interventions from experienced, certified physical therapists: spinal mobilization with leg movement or spinal extension–oriented mobilization. Both techniques were designed to improve spinal alignment and enhance neural mobility [20, 30]. The interventions were administered twice per week over a 2‐week period, totaling four treatment sessions.

### 2.4. Statistical Analysis

Descriptive statistics for the demographic characteristics of the participants were presented using means and standard deviations for continuous variables and frequencies for categorical variables. The primary aim of the statistical analysis was to evaluate the responsiveness of the outcome measures employed in the study. Responsiveness refers to the ability of a measure to detect change over time, particularly in response to an intervention. In this study, two approaches were used to assess responsiveness: the calculation of change scores and effect size (ES).

The change score was computed by subtracting the baseline (pretest) value from the posttest value. This score represents the difference observed following the intervention period, with higher values indicating a greater degree of change and, consequently, higher responsiveness. The ES is calculated by dividing the mean change score by the standard deviation of the baseline values [17]. Spearman’s rank correlation coefficient was employed to assess the relationships between change scores (baseline and at the post‐2‐week time point) for pain, clinical test results, and disability. The interpretation is as follows: ES below 0.2 is considered small, 0.5 is moderate, and 0.8 is large. The interpretation of the correlation coefficient is as follows: *r* below 0.3 is a weak correlation, *r* = 0.3–0.69 is a moderate correlation, and *r* ≥ 0.7 is a strong correlation [31]. The significance was set at *p* value < 0.05.

## 3. Results

Data from 32 participants were collected and analyzed statistically. All patients were diagnosed with LDH. The most prevalent level of herniation was L4‐L5, occurring in 16 patients (50%). No sensory or motor impairments were reported. Based on the inclusion criteria, patients with Grade 1 or Grade 2 LDH were recruited. The mean age of the participants was 42 years. The sample included an equal distribution of sexes, with 16 males and 16 females. Demographic characteristics of the participants are summarized in Table 1.

**TABLE 1:** Demographic data of the subjects.

Characteristics	Summary, *n* = 32
Age (years)	42.47 ± 9.53
Sex: male/female	16/16
Height (centimeters)	164.22 ± 6.56
Weight (kilograms)	65.80 ± 10.28
Body mass index, (kilograms/meter^2^)	24.34 ± 2.92
Affected side: right/left	13/19
Levels of LDH	
L2‐L3	1
L3‐L4	5
L4‐L5	16
L5‐S1	12
Symptom onset	
Acute phase	14
Subacute phase	18
Chronic phase	0

Abbreviation: LDH = lumbar disc herniation.

Table 2 presents the baseline and follow‐up data, including postimmediate, post‐1‐week, and post‐2‐week assessments, for pain intensity (at rest and at worst), clinical tests, and disability levels. Table 3 summarizes the change scores and ES for all outcome measures. Regarding pain at rest, the mean change scores were −1.2 cm immediately postintervention, −2.3 cm at 1‐week follow‐up, and −3.6 cm at 2‐week follow‐up, indicating a progressive reduction in pain over time. The corresponding ES values for pain at rest were greater than 1.0 at every time point, suggesting large treatment effects. The change scores and ES values for pain at worst followed a similar trend to those observed for pain at rest, but the ES for pain at worst was larger (ES = 1.5) at postimmediately compared to pain at rest.

**TABLE 2:** Baseline and follow‐up data of pain, clinical tests, and disability.

Parameters	Baseline	Postimmediately	Post‐1‐week	Post‐2‐week
Visual analog scale				
Pain at rest (centimeters)	5.9 ± 1.0	4.7 ± 0.9	3.6 ± 0.8	2.3 ± 0.8
Pain at worst (centimeters)	6.4 ± 1.2	4.6 ± 1.0	3.6 ± 1.0	2.1 ± 0.9
Clinical tests				
Straight leg raise (degrees)	54.2 ± 19.4	57.4 ± 20.1	64.6 ± 19.4	72.0 ± 15.0
Five‐meter walk test (seconds)	5.4 ± 1.0	5.2 ± 0.8	4.9 ± 0.8	4.5 ± 0.6
Disability				
MODI total score (%)	32.2 ± 15.3	—	—	16.3 ± 10.2

Abbreviation: MODI = the Modified Oswestry Disability Index.

**TABLE 3:** Change score and effect size of pain, clinical tests, and disability.

Parameters	Postimmediately		Post‐1‐week		Post‐2‐week	
	Change score	Effect size	Change score	Effect size	Change score	Effect size
Visual analog scale						
Pain at rest (centimeters)	−1.2	1.2	−2.3	2.3	−3.6	3.6
Pain at worst (centimeters)	−1.8	1.5	−2.8	2.3	−4.3	3.6
Clinical tests						
Straight leg raise (degrees)	3.2	0.2	10.4	0.5	17.8	0.9
Five‐meter walk test (seconds)	−0.2	0.2	−0.5	0.5	−0.9	0.9
Disability						
MODI total score (%)					−15.9	1.0

Abbreviation: MODI = the Modified Oswestry Disability Index.

For clinical test outcomes, the ES values showed a gradual increase from the postimmediate to the post‐2‐week assessment. The ESs ranged from 0.2 to 0.9, indicating small to large improvements. These patterns were consistent across both the SLR and the 5MWT.

Regarding disability, the MODI total score demonstrated a change of −15.9%, with an associated ES of 1.0. This result reflects a substantial improvement in functional disability over the 2‐week intervention period.

Correlations between change scores for pain, clinical test results, and disability were generally nonsignificant. The only exception was a moderate positive correlation between changes in pain at rest and MODI (*r*_*s*_ = 0.408, *p* value = 0.021) (Table 4).

**TABLE 4:** Correlations of change score of pain, clinical tests, and disability.

Variables	Statistic parameters	Pain at worst	Straight leg raise	Five‐meter walk test	MODI
Pain at rest	Spearman *r*	0.301	−0.016	−0.050	**0.408**
	*p* value	0.094	0.932	0.786	0.021**^∗^**
Pain at worst			−0.004	0.029	0.167
			0.981	0.873	0.360
Straight leg raise				−0.027	−0.154
				0.881	0.400
Five‐meter walk test					0.184
					0.314

*Note:* Bold values represent significant correlation.

^∗^Significant difference at *p* < 0.05.

## 4. Discussion

The study aimed to investigate the responsiveness of clinical outcome measures in patients with RLBP on pain intensity (at rest and at worst), SLR, 5WMT, and disability (MODI) over a 2‐week interval between baseline and posttest assessments.

The findings of our study align with the extensive literature; both narrative and systematic reviews underscore the efficacy of nonsurgical physical therapy for LDH with radiculopathy. Our results further support these interventions as a primary treatment strategy to reduce pain and disability when no red flags are present [32–34]. We measured pain intensity at rest and at worst, since the patients with RLBP had pain at rest as a resting symptom. The results found that the baseline pain intensity for both at rest and at worst was moderate, 5.9 and 6.4 cm on VAS, respectively. Change scores that exceeded the MCID of 2.25 [23] were detected after 1 and 2 weeks. The ES values for both pain at rest and pain at worst were large since postimmediately until after 2 weeks. Compared to acute NSLBP [19], the ES at 2 weeks after the intervention program for RLBP was greater, 3.6 versus 1.57. This highlights the efficacy and essential role of therapeutic programs aimed at alleviating pain intensity in patients with RLBP. Therapeutic interventions are particularly critical, as they contribute not only to the restoration of spinal alignment and functional mobility but also to the protection and management of neural structures. Comprehensive care that addresses both the musculoskeletal and neural components is therefore fundamental in the rehabilitation process for individuals with RLBP [9, 20, 30].

Clinical tests, SLR, and 5MWT showed similar trends. The improvement for both SLR and 5MWT was gradual and continuous since the ES was from 0.2 (small) at postimmediately to 0.5 (moderate) at 1 week and 0.9 (large) at 2 weeks after the baseline. The MDC for SLR is 5.7° [24]. Significant changes exceeding the MDC were observed at both 1 week and 2 weeks following the baseline measurement. The MCID for 5MWT is 0.5 s [25]. Significant changes exceeding the MCID were observed at both 1 week and 2 weeks following the baseline measurement. This finding suggests that patients with RLBP may experience significant improvements in neurodynamic function and walking speed as early as 1 week following the initial therapeutic interventions. However, as these two variables have not been definitively validated for measuring longitudinal improvement in patients with lumbar radicular pain, their use should be approached with caution. They are not recommended as standalone measures for evaluative purposes.

Regarding disability, the MODI was used to evaluate the disability level. The change score for the MODI was −15.9% at 2 weeks postbaseline, exceeding both the MCID and the MDC thresholds of 9% [23, 28]. Furthermore, the change in MODI scores demonstrated a significant correlation with pain levels at rest, suggesting that improvements in functional disability are closely mirrored by a reduction in resting pain. This implied that the decrease in disability level could be expected in patients with RLBP at 2 weeks postbaseline. Similarly to patients with acute NSLBP [19], the change score was −16.2%, exceeding the MDC and MCID, and the ES was 0.9 (large), at 2 weeks postbaseline. However, for patients with chronic NSLBP [18], the change score was only −5%, and the ES was 0.4 (small). This finding highlights that acute cases, whether classified as RLBP or NSLBP, have the potential for rapid and substantial recovery. Therefore, clinicians should emphasize providing immediate and effective care for patients presenting with an acute onset.

Moderate correlations were detected between pain at rest and pain at worst, and pain at rest and MODI scores. This correlation represented the convergent validity between pain at rest and pain at worst and MODI. However, the correlations were low between pain at rest and SLR and 5MWT. This might indicate the divergent validity of these 3 clinical outcomes in different aspects of the clinical manifestation of LDH. The different construct might be due to the fact that pain is a sensory experience, often driven by chemical inflammation around the nerve root in LDH. In contrast, the SLR is a mechanical provocation test; the SLR might not be as restricted as the pain level would suggest. In addition, the 5MWT was a functional capacity, which could be affected by several factors such as compensatory strategies and fear avoidance. Therefore, the SLR or 5MWT was not suggested to be used solely as an indicator of recovery. Future studies are needed to confirm the clinical utility of these measures before a formal recommendation for their implementation can be issued.

Clinical implications, pain intensity (at rest and at worst), SLR, 5MWT, and disability level were responsive to therapeutic interventions within 2 weeks in patients with RLBP. Furthermore, progressive pain reduction was observable weekly. This finding supports the potential for continuous clinical monitoring throughout the early phase of treatment. However, based on the magnitude and consistency of observed changes, the optimal time point for assessing all measured clinical outcomes appears to be 2 weeks following the initiation of therapeutic interventions.

Limitations of the study: the data were obtained from only patients with acute and subacute RLBP. The future study could explore the responsiveness of the clinical outcome measures in chronic RLBP for better clinical comprehension. The longer period of follow‐up should be considered to understand the nature of RLBP symptoms and the sustainability of therapeutic interventions. In addition, these promising results may be attributed to the specialized clinical expertise of the certified physical therapists involved in the study. However, this reliance on high‐level practitioner skill may limit the generalizability of the findings to broader clinical settings where therapist experience varies.

## 5. Conclusion

This study demonstrated that all clinical outcome measures, namely, pain intensity (at rest and at worst), SLR, 5MWT, and disability level, were responsive to therapeutic interventions within a 2‐week period in patients with acute and subacute RLBP. This responsiveness was evidenced by large ESs, indicating meaningful clinical change. Notably, both pain at rest and pain at worst exhibited large ES even after a single session of intervention, suggesting that these parameters are particularly sensitive to immediate therapeutic effects.

## Author Contributions

Halidadjiyah, Prasert Sakulsriprasert, and Wunpen Chansirinukor contributed equally to this study.

## Funding

No funding was received for this study.

## Conflicts of Interest

The authors declare no conflicts of interest.

## Data Availability

Data are available from the corresponding author upon reasonable request.
